# The use of hyperbaric oxygen therapy in acute hearing loss: a narrative review

**DOI:** 10.1007/s00405-019-05469-7

**Published:** 2019-05-20

**Authors:** A. B. Bayoumy, J. A. de Ru

**Affiliations:** 1 Department of Otorhinolaryngology, Central Military Hospital Utrecht, Ministry of Defense, Lundlaan 1, 3584 EZ Utrecht, The Netherlands; 2 Department of Otorhinolaryngology, University Medical Center Utrecht, Utrecht, The Netherlands

**Keywords:** Acute hearing loss, Acute acoustic trauma, Idiopathic sudden sensorineural hearing loss, Hyperbaric oxygen therapy

## Abstract

**Introduction:**

Acute hearing loss can have a major impact on a patient’s life. This holds true for both acute acoustic trauma (AAT) and idiopathic sudden sensorineural hearing loss (ISSHL), two devastating conditions for which no highly effective treatment options exist. This narrative review provides the rationale and evidence for HBOT in AAT and ISSHL.

**Methods:**

Narrative review of all the literature available on HBOT in acute hearing loss, studies were retrieved from systematic searches on PubMed and by cross referencing.

**Discussion:**

First, the etiological mechanisms of acute hearing loss and the mechanism of action of HBOT were discussed. Furthermore, we have provided an overview of 68 studies that clinically investigated the effect of HBOT in the last couple of decades. For future studies, it is recommend to start as early as possible with therapy, preferably within 48 h and to use combination therapy consisting of HBOT and corticosteroids.

**Implications for practice:**

HBOT has been used quite extensively for acute hearing loss in the last couple of decades. Based on the amount of studies showing a positive effect, HBOT should be discussed with patients (shared decision making) as optional therapy in case of AAT and ISSHL.

## Introduction

Acute hearing loss can have a major impact on a patient’s life. This holds true for both acute acoustic trauma (AAT) and idiopathic sudden sensorineural hearing loss (ISSHL), two devastating conditions for which no highly effective treatment options exist [1, 2].

In the US Army over a 2-year period, more than 882 hearing impairments were caused by AAT, and the incidence of ISSNL in the USA was estimated to be 27 per 100,000, which corresponds to more than 66,000 new cases per year [3, 4].

The amount of hearing loss in AAT can vary between individuals and is based on the amount and duration of noise exposure [5]. For ISSHL, the amount of hearing loss can vary from 30 dB at three frequencies, to even losses of 120 dB at more frequencies [6]. For smaller losses, the natural course might be favorable due to the—albeit limited—repair capacity of the cochlea; however, in profound cases the chance of complete recovery is rather low. Furthermore, in case of AAT a temporary threshold shift can occur, which makes it difficult to immediately evaluate the amount of damage and subsequently the amount of recovery [7]. The definitions of ‘complete recovery’, ‘good recovery’, and other terminology were not used unambiguously in literature making comparison between studies or pooling of results difficult.

The cochlea is an organ with an impressive activity; therefore, it is always dependent on adequate oxygen levels in the blood [8]. However, because of the protected location of the cochlea in the temporal bone, blood supply is limited [9]. One of the mechanisms playing a possible role in both AAT and ISSHL is of vascular origin: lack of oxygen. For AAT, Misrahy et al. [10] found evidence for the vascular theory in 1958. They reported cochlear endolymphatic hypoxia during noise exposure and interpreted this as caused by a reduced cochlear blood flow. Numerous studies have later demonstrated signs of reduced blood flow due to vasoconstriction and cochlear hypoxia in AAT [11–16]. In ISSHL, this vascular etiology was described by Ruben et al. [17]. Other authors since then have also found a vascular etiology for sudden deafness [18–24].

All this has led to the experimental use of vasoactive drugs and other therapies that tried to nullify the hypoxia. Unfortunately, none of those therapies was proven effective [25]. Already in 1956, Boerema, the father of modern hyperbaric medicine, was the first to describe the use of hyperbaric oxygen therapy (HBOT) in combination with cardiac surgery [26]. During HBOT, 100% oxygen is inhaled at a pressure of about 200–300 kPa, in sessions of 2–3 h per day, for about 10–25 days. This is almost sufficient to supply the resting total oxygen requirements of many tissues without a contribution from oxygen bound to hemoglobin. HBOT may increase the oxygen load to the cochlea, eradicating hypoxia. Despite the above-mentioned rationale for therapy, not everyone is convinced of the efficacy of HBOT. This is probably because of the fact that most physicians are not very well acquainted with this therapy and certainly not for this indication. However, according to the Cochrane Library, no other specific drug therapy has been documented to be proven effective against hearing loss in case of AAT or ISSHL. In recent years, HBOT has been used for AAT and ISSHL by many colleagues; however due to the lack of randomized controlled trials, it is still unclear what the role of HBOT should be in the treatment protocol of these two types of acute hearing loss [27]. This narrative review provides the rationale and evidence for HBOT in AAT and ISSHL.

## Etiological mechanism of hearing loss

### The first reports on hypoxia and hyperbaric oxygen therapy

Some 40 years ago, the first steps were made in understanding the theoretical framework for HBOT in inner ear disease. First, it was reported that in conditions of low oxygen, the cochlear potential diminished and failed to reappear after restoration of the blood supply [28]. In a lower oxygenated state, the cochlear potential was found to be 20 mV more negative than in the oxygenated state [29].

Noise induces a decline of oxygen pressure in the perilymph of the scala tympani by more than half [30].

Another report by Scheibe et al. found that oxygenation of the cochlear perilymph decreased by 20% during high-intensity acoustic exposure (125 dB) [31]. Also, in case of ISSHL, it was found that the perilymphatic oxygen tension was low [32].

In a review on seven experimental studies performed by Axelsson and Dengerink, it was concluded that noise induces a reduction in cochlear blood flow [33–41]. In the following years, other authors found the same conclusion. Yamane et al. reported blood stagnation in the strial capillaries, leading to strial dysfunction, after acoustic trauma in guinea pigs [16]. In 1992, Quirk et al., observed capillary vasoconstrictions and decreased red blood cell velocity after noise exposure (110 dB) with intravital microscopy in vivo [13].

Due to all the experimental findings that suggested a correlation between low oxygen levels in the inner ear and the pathogenesis of hearing loss, experiments with the use of HBOT were performed.

In 1982, Lamm et al. assessed the effects of HBOT in an experimental AAT model in guinea pigs. They showed an alleged preventive effect of HBOT in 14 out of 26 guinea pigs [42].

Hu et al. reported that HBOT can reduce the noise-induced threshold shift and decrease cochlear damage during chronic noise exposure in guinea pigs [43].

### Mechanically induced hair cell damage

Intense noise produces mechanical damage to the cochlea, which directly leads to disruption of the hair cell stereocilia [44, 45]. Threshold shifts (auditory-nerve responses) are correlated with the amount of damage or loss in hair cells after acoustic trauma [46]. Pilgramm et al. demonstrated in an animal model that 60 h after acoustic trauma, the number of inner ear sensory cells that had suffered morphological damage was lower in the group receiving HBOT than in the group without HBOT [47]. Kuokkanen (1997) and (2000) showed a lesser amount of threshold shift and fewer missing hair cells among rats that were treated with HBOT following 60 shots (162 dB) with an assault rifle [48, 49].

Colombari et al. found in an animal experiment with acute acoustic damage that the number of injured cochlear outer hair cells decreased and that their functionality improved after HBOT [50].

### Reactive oxygen species

Due to reduction of the blood flow, reduced oxygen supply causes the phosphorylation process within the mitochondria to become inefficient. This causes the formation of reactive oxygen species (ROS) as a by-product of metabolism. In 1995, Yamane et al. published a study on the formation of free radicals within the inner ear directly after acoustic trauma [51]. Ohlemiller et al. found a nearly fourfold increase in ROS levels in animals after noise exposure compared to non-exposed animals [52]. Ohinata et al. observed increased 8-isoprostane levels, which serve as an accurate marker for free radical-catalyzed lipid peroxidation [53] during noise exposure and these levels diminished directly after the termination of exposure [54]. Furthermore, ROS play an important role in the local inflammation caused by noise-induced damage. After ROS formation in the cochlea, interleukin-6 and tumor necrosis factor alpha were found to be produced locally. These pro-inflammatory cytokines can themselves produce damage to the cochlea [55–57]. Also, ROS formation activates the c-Jun N-terminal kinase (JNK) signaling pathway [58]. The JNK signaling pathway and mitogen-activated protein kinase (MAPK) pathways are increased after acoustic trauma; these pathways mediate programmed apoptosis of outer hair cells [59]. Han et al. reported that apoptosis inducing factor (AIF) and endonuclease G (endoG) were found in the nucleus of apoptotic hair cells [60].

Following ischemia, the blood flow may recover and consequently the availability of oxygen rises, and as a result of this reperfusion more ROS may be formed [51].

Therefore, safety concerns have been raised for HBOT, since the higher availability of oxygen within the inner ear may further boost the formation of ROS. Arslan et al. did an experiment in which they measured pro-inflammatory cytokines and hearing levels in rats exposed to acoustic trauma. They found that treatment with HBOT in the first 3 h after acoustic trauma resulted in significantly higher IL-1β levels compared to the control group. The group in which HBOT was started within 24 h after acoustic trauma had no significantly different pro-inflammatory mediators compared to the control group, and had significantly better post-treatment auditory brain stem responses compared to the other groups [61].

Kahraman et al. exposed 16 rats to noise of 110 dB for 1 h. All rats, except those in the control group, were treated with immediate HBOT within 1 h of noise exposure. They obtained lower hearing gains in the HBOT groups compared to the control group. However, in the group in which HBOT was combined with corticosteroids, significant recoveries were found. This study highlights the negative effects of the extremely early initiation of HBOT and the importance of combining HBOT and corticosteroids, in terms of better recovery [62]. In clinical practice, however, it would not be possible to initiate HBOT on such a short notice. Thus, HBOT combined with corticosteroids can be deemed safe when used at least 24 h after the onset of hearing loss.

Moreover, it has been found by Hamernik et al. that the hair cell death after noise exposure is relatively small, but that the loss of hair cells increases over a period of 2–30 days after exposure [63]. In line with this, Yamashita et al. found a correlation with the delayed formation of ROS and the progressive hair cell loss, which stabilized after 2 weeks [64].

Therefore, based on the evidence provided above, HBOT may play a very important role in the early prevention of further damage to the hair cells caused by the reduction of cochlear blood flow and formation of free radicals. But, a reduction of the inflammatory reaction might also be necessary. Takemura et al. indeed found protective effects of dexamethasone infusion in guinea pigs after acoustic trauma, attributed to the attenuation of the threshold shift and increase in hair cell preservation [65].

Extensive reviews on the etiological mechanisms of AAT have been performed by Kurabi (2017), Wong (2015), Shi (2011) and Henderson (2006) [9, 66–68].

## Clinical use of hyperbaric oxygen therapy

Since the late 1960s, HBOT has been experimentally used in clinic for AAT and ISSHL as well. Appaix [69] and Lamm [70] are credited for their pioneering work on the role of HBOT in these two conditions. In 1976, De Heyn and van Opstal treated two series of patients following AAT, one group with vasodilators and the other with a combination of vasodilators and hyperbaric oxygen. They found better results in the patients with the combination therapy [71]. Vincey reported on the rationale for HBOT inner ear disease in 1978 [72]. In 1979, Goto et al. performed a study on 91 patients suffering from ISSHL, of whom 20 patients were treated with a combination of vasodilators, corticosteroids, vitamins, stellate ganglion block and HBOT. All patients in this treatment group had improved hearing (> 10 dB) and 40% had recovered completely [73].

In 1981, Demaertelaere and van Opstal published a study in the Dutch language on the treatment of AAT with HBOT [74]. Vavrina and Muller published a similar study in French in 1995 [75]. Pilgramm and Schumann published a series of 122 soldiers following AAT [76]. These patients were randomly allocated to different treatment groups. The authors of these studies were positive about the results of HBOT in AAT patients. Regarding AAT, Winiarski et al. found significant differences on 4, 6 and 8 kHz when HBOT was started within 5 days in patients suffering from AAT [77]. Three years later, in 2008, Ylikoski et al. published the results of a study in which HBOT monotherapy was compared to normobaric oxygen therapy in a cohort of AAT patients, who were exposed to firearm shooting. They reported significantly higher hearing gains in the HBOT group compared to the normobaric group (69.3 vs 56.2%, *P* < 0.001) [78].

Since 1998, when Lamm et al. [79] and Nakashima et al. [80]. published positive clinical results of HBOT for ISSHL in the English language, a storm of articles was published in the first decade of this century, by Aslan et al., Racic et al., Topuz et al., Narozny et al., Horn et al., Desloovere et al., Dundar et al., Fujimura et al. and Muzzi et al., confirming the earlier outcomes [81–89].

Negative results were published by Kestler et al. [90]. in a German study in 2001, and by Satar et al. [91]. who found that the hearing gains in the HBOT and control group were 35.5 vs 37.0 dB (*P* = 0.754). They also found that the hearing improvement (gain > 10 dB) was 60% in the HBOT group and 76.4% in the control group (*P* = 0.364). But, when calculating the relative gain using the data mentioned in their manuscript, it was 52.1% for the HBOT group and 45.0% for the control group.

### Steroid therapy

As described above, both in case of AAT and ISSHL, an inflammatory response occurs as part of the pathophysiology [66, 92, 93]. Therefore, it is deemed necessary to stop the inflammatory reaction. The first randomized controlled study with corticosteroids for ISSHL was performed by Wilson et al. in 67 patients. They found that patients treated with corticosteroids had statistically higher recovery than the placebo group (61% vs 32%). Complete recovery in their study was defined as: “hearing within 10 dB of pre-hearing loss speech reception or averaged pure tone score. Partial recovery of hearing to 50% or more of the pre-hearing loss speech reception score or average pure tone score” [94].

Corticosteroids increase oxygen consumption by mobilizing amino acids for gluconeogenesis and by altering glucose utilization by oxygen-consuming mechanisms [95]. This higher oxygen consumption may enhance the decline of the partial oxygen pressure in the perilymph, as observed in animals exposed to noise and treated with corticosteroids [96]. Therefore, adding HBOT to corticosteroids might be indicated on theoretical grounds, so one might ask: *does combining corticosteroids and HBOT potentiate the effect of corticosteroids monotherapy?* In an experimental animal study on AAT, D’Aldin et al. found that combination therapy in animals with HBOT and corticosteroids significantly improved threshold shifts compared to the control group receiving only corticosteroids [97]. In the same year, similar results were found by Lamm and Arnold who concluded that the combination of HBOT and prednisolone had a better therapeutic effect on noise-induced hearing loss in guinea pigs than HBOT or prednisolone as monotherapy [12].

Also Fakhry et al. stated that combined HBOT and corticosteroid therapy provided more protection in vivo from acoustic trauma, especially when started within 1 day following the exposure [98].

During the last decade, even more clinical evidence suggested that combination therapy HBOT and corticosteroids results in better recovery than both treatments alone. For example Alimoglu et al. [99] obtained higher hearing gains and higher response to therapy in ISSHL patients treated with combination therapy compared to those therapies as individual entities, while Capuano et al. [100] reported that the combination therapy with HBOT and intravenous corticosteroids had significantly higher mean gains at 0.5, 1, 2 and 4 kHz compared to both HBOT and intravenous corticosteroids as monotherapies in patients with ISSHL. Still, more studies can be traced in which HBOT was combined with corticosteroids [73, 75, 81, 83, 88, 91, 99–127].

### Recent findings

In 2019, Bayoumy et al. reported the positive effects of HBOT and corticosteroids compared to corticosteroid monotherapy in a cohort of AAT patients. Absolute (21.3 dB vs 11.6 dB) and relative (57.6% vs 31.4%) hearing gains were statistically significantly in favor of HBOT therapy. They found a higher percentage of patients recovering to an acceptable level for the Dutch Armed Forces in the HBOT group [126].

In 2018, six studies investigating the effect of HBOT and corticosteroids in ISSHL were published. Interestingly enough, these latest studies have a higher evidential level due to better adherence to methodological regulations as compared to many of the previous reports.

Chi et al. treated 30 patients with only medical therapy (pentoxifylline, dextran and corticosteroids) and 30 patients with conventional therapy plus additional HBOT. In both groups, therapy was initiated early, on average within 4 days. Complete hearing recovery was significantly better in the HBOT group after 180 days (27% vs 10%, *P* = 0.043) [121].

Almosnino et al. investigated two groups of patients receiving intratympanic or oral corticosteroids (control) compared to the same treatments with additional HBOT. They observed hearing gains of 17.9 dB in the HBOT group, and 15.0 dB in the control group. They concluded that HBOT did not show a beneficial effect over therapy with corticosteroids. However, HBOT was initiated till even as much as 3 months after hearing loss [122].

Krajcovicova et al. performed a prospective study in 68 patients suffering from ISSHL, in which 47 were treated with additional HBOT. They found a hearing improvement rate (defined as hearing ≥ 10 dB) of 61.7% in the HBOT group, compared to only 28.6% in the control group. The average hearing gain in the HBOT group was 20 dB, whereas in the control group it was 8.5 dB. Treatment was initiated within 7 days [123].

Cho et al. compared one group treated with systemic and intratympanic corticosteroids to another group with the same treatments plus HBOT. They found significantly higher word discrimination scores in favor of the HBOT group (66% vs 13%, *P* = 0.029). The absolute hearing gains between the HBOT and control group was not significantly different (47.3 dB vs 37.7 dB, *P* > 0.05) [124].

Khater et al. treated 11 patients with medical therapy (systemic corticosteroids, intratympanic corticosteroids, antiviral therapy) and 11 patients with medical therapy plus HBOT. The absolute hearing gain was significantly higher in the HBOT group compared to the control group (54.8 dB vs 43.8 dB, *P* = 0.0014). Regarding recovery, 8 out 11 (73%) of HBOT patients showed total improvement at all frequencies, while only 6 out of 11 (46%) showed total improvement in the control group; however, this difference was not significant (*P* > 0.05) [125].

Hosokawa et al. compared three different treatment strategies: HBOT and systemic corticosteroids vs intratympanic corticosteroids and systemic corticosteroids vs systemic corticosteroids alone. They mentioned that the complete recovery rates were 26.1% in the HBOT group, 8.6% in the combined corticosteroids group and 6.3% in the systemic corticosteroids group. The overall recovery was 78.3% for the HBOT group, 48.6% for the combination corticosteroids group and 32.5% for the systemic corticosteroids group. Treatment was initiated within 4.5 days after onset of hearing loss [127].

Thus, it seems, based on recent evidence, that addition of HBOT to corticosteroids may be beneficial, especially when initiated early, in both AAT and ISSHL. Furthermore, Table 1 shows the outcomes of the performed studies in AAT and ISSHL performed in the last decades.

**Table 1:** The percentages of hearing gain and recovery in studies that compared HBOT against medical therapy (MT) in ISSHL

Author	Year	Dx	Study design	Medical therapy	Time till initiation of HBOT	Salvage?	Absolute hearing loss (dB)	Relative hearing loss (%)^a^	Complete recovery (%)	Remarks
Bayoumy [126]	2019	AAT	RS	SS vs SS + HBOT	4.4	No	HBOT: 23.5 MT: 12.5	HBOT: 57.6 MT: 31.4	HBOT: 61.1 MT: 8.3	Early treatment (within 7 days) was significantly better than after 7 days in the HBOT group
Hosokawa [127]	2018	ISSHL	RS	SS vs ITS + SS vs HBOT + SS	4.5	No	NA	NA	HBOT: 26.1 SS: 6.3	Overall recovery in the HBOT group was 78.3% vs 32.5% in the SS group (*P* < 0.001)
Khater [125]	2018	ISSHL	RS	MT: SS + ITS + Acyclovir vs HBOT + MT	≤ 7	No	HBOT: 54.8 MT: 43.8	HBOT: 76.1 MT: 60.9	HBOT: 72.7 MT: 45.5	Early treatment (within 7 days) with combination therapy showed better results
Cho [124]	2018	ISSHL	PS	SS + ITS vs SS + ITS + HBOT	4.1	No	HBOT: 47.3 MT: 37.7	HBOT: 65.7 MT: 40.8	HBOT: 36.7 MT: 16.7	Significantly better results in favor of HBOT on 0.5 and 1 kHz and word discrimination score
Krajcovicova [123]	2018	ISSHL	PS	MT: SS, BH, Agapurin vs MT + HBOT	≤ 7	No	HBOT: 20.0 MT: 11.5	HBOT: 61.7 MT: 28.6	HBOT: 6.4 MT: 0.0	Better results for HBOT with regard to hearing gains and complete recovery (> 10 dB hearing gain)
Almosnino [122]	2018	ISSHL	RS	ITS or SS (presalvage) ITS vs ITS + HBOT	29.1	Yes	HBOT: 17.9 MT: 15.0	HBOT: 22.9 MT: 22.0	NA	No difference between combination therapy and ITS alone as salvage therapy
Chi [121]	2018	ISSHL	PS	MT: SS + PF + DX vs MT + HBOT	4.2	No	NA	NA	HBOT: 26.7 MT: 10.0	Better hearing recovery in the HBOT group (*P* = 0.043)
Sun [139]	2018	ISSHL	RS	ITS vs HBOT vs control	5.3 + 10	Yes	NA	NA	NA	No difference between the three groups
Xie [120]	2018	ISSHL	RS	SS, blood flow promoters + vitamin B complex + HBOT	7.8	No	NA	NA	19.7	Prognostic factors: initial hearing loss, vertigo, early onset of HBOT (better), number of HBOT treatments, profound hearing loss, flat and ascending audiogram
Van Haesendonck [119]	2018	AAT	PS	SS + HBOT	4.1	No	NA	NA	NA	Significantly less TFI, VASM SNHL, tinnitus and subjective hearing loss after HBOT
Ricciardello [118]	2017	ISSHL	RS	MT: IVS + SS + pantoprazole + glycerol (10%), enoxaparin sodium vs MT + HBOT	7.2	No	NA	NA	HBOT: 44.1 MT: 31.8	Age, time since onset of hearing loss, audiometric type and vertigo were significantly correlated with recovery
Tasdöven [150]	2017	ISSHL	RS	SS vs SS + ozone (MT) vs HBOT	NA	No	NA	NA	HBOT: 11.5 MT: 17.6 SS: 22.2	More response to treatment (gain > 15 dB) in the MT group compared to corticosteroids only and HBOT (82.4 vs 50.8 vs 61.5%)
Hosokawa [138]	2017	ISSHL	RS	SS (presalvage) HBOT vs no therapy	< 30	Yes	NA	NA	HBOT: 9.6 MT: 6.2	Age, initial hearing level and time from onset of symptoms till therapy were all not significantly predictive for hearing recovery The percentage of no change was lower in the HBOT group (53.9%) compared to the control group (67.5%)
Hosokawa [131]	2017	ISSHL	RS	ITS + HBOT	≤ 7 or > 7	No	NA	NA	25.5	The improvement rate was significantly higher if treatment was within 7 days (82.2%) of onset of symptoms compared to > 7 days (42.7%, *P* < 0.001). Younger patients (≤ 60 years) had significantly better improvement rate
Ajduk [134]	2017	ISSHL	RS	SS (presalvage) HBOT vs no therapy	23 + 11.4	Yes	NA	NA	NA	Significant hearing improvements at 0.25; 0.5 and 8 kHz after HBOT. Significant hearing gains on all frequencies when hearing loss was > 60 dB
Lamm [152]	2016	ISSHL	CR	ITS + HBOT	NA	Yes	NA	NA	NA	Beneficial effect of salvage HBOT
Akil [117]	2016	ISSHL	RS	SS + HBOT	4.1	No	Uni: 22.1 Bi: 8.2	Uni: 32.0 Bi: 12.6	NA	Unilateral vs bilateral ISSHL No effect of HBOT in bilateral hearing loss
Gülüstan [116]	2016	ISSHL	RS	ITS vs HBOT	11.3	Yes	HBOT: 12.8 ITS: 20.2	HBOT: 21.1 ITS: 28.3	HBOT: 22.2 ITS: 20.0	No significant difference between HBOT and ITS
V/d Wal [153]	2016	ISSHL	CR	HBOT monotherapy	333 min	No	NA	NA	41.0	HBOT resulted in hearing improvement in 80% of patients
Karatop-Cesur [132]	2016	ISSHL	RS	HBOT monotherapy	6.8	No	NA	NA	53.8	Better outcome with early treatment response (≥ 10 dB gain in the first week)
Sherlock [135]	2016	ISSHL	RS	HBOT monotherapy	13	No	23	33.3	NA	Younger age (< 60), hearing loss > 60 dB and early HBOT initiation were positive prognostic factors
Živaljević [154]	2016	ISSHL	RS	HBOT monotherapy	NA	No	24.9	65.45	55.0	Study recommended HBOT as primary treatment for ISSHL
Psillas [115]	2015	ISSHL	RS	IVS + piracetam (presalvage) HBOT vs no therapy	24	Yes	HBOT: 14.9 MT: 2.6	HBOT: 21.8 MT: 3.7	HBOT: 6.6 MT: 0.0	No difference between patients within or later than 20 days. Authors suggest salvage HBOT after therapy failure
Pezzoli [114]	2015	ISSHL	PS	IV mannitol (18%) + IV betamethasone (presalvage) Dexamethasone vs dexamethasone +HBOT	9.7 days	Yes	HBOT: 15.6 MT: 5.0	HBOT:21.6 MT: 8.2	HBOT: 4.3 MT: 0.0	Significant hearing improvement after HBOT for patients who failed primary therapy
Capuano [100]	2015	ISSHL	RS	IVS vs HBOT vs IVS + HBOT	< 90	No	NA	NA	HBOT: 58.0 IVS: 20.0	Significant more hearing gain when initial hearing ≥ 91 dB. Significant higher gains when therapy started within 14 days. Patients responding to therapy highest in combination group (84%) compared to HBOT and IVS (70% and 68%)
Edizer [113]	2015	ISSHL	RS	SS vs SS + LMWH vs SS + HBOT vs SS + LMWH + HBOT (MT)		No	NA	NA	HBOT: 31.2 MT: 31.2	Time till treatment > 10 days had a worse prognosis. Age, degree of hearing loss and hypertension affect the prognosis of ISSHL
Naiboğllu [112]	2015	ISSHL	PS	SS + HBOT vs SS + HBOT + ITS	≤ 3	No	23.9	30.8	30	Only beneficial effect of ITS in profound hearing loss
Attanasio [111]	2015	ISSHL	PS	HBOT + ITS	< 15	No	29.4	31.9	59.3	No difference between HBOT treatment once or twice a day
Yildirim [110]	2015	ISSHL	RS	IVS + IV piracetam + HBOT	Three groups ≤ 7, (8-14), > 14	No	18.9	34.6	NA	Treatment within 14 days significantly better than after 14 days
Guha [155]	2015	ISSHL	PS	HBOT monotherapy	NA	No	17.6	22.1	54.9	
Bonfort [109]	2014	AAT	RS	IVS, PF and HBOT	< 24 h	No	18.3	53.8	NA	
Yang [108]	2013	ISSHL	RS	ITS (MT) vs HBOT vs ITS + HBOT vs control	5.2	Yes	HBOT: 22.5 MT: 18.9	HBOT: 23.1 MT: 20.0	HBOT: 68.4 MT: 48.6	Significant difference in recovery rate (*P* = 0.018) in favor of combination therapy Significant higher gains on 0.25; 0.5, 2 kHz for combination therapy
Cvorovic [137]	2013	ISSHL	RCT	ITS vs HBOT	< 4 weeks	Yes	NA	NA	NA	Significant hearing gain in favor of HBOT on 2 kHz (*P* < 0.05) Higher hearing gains in ITS group when initial hearing loss ≥ 81 dB
Suzuki [107]	2012	ISSHL	RS	SS + ITS vs HBOT + SS	6.1	No	HBOT: 26.2 MT: 27.0	HBOT: 51.6 MT: 50.2	HBOT: 29.3 MT: 21.6	Significant higher recovery rate in favor of MT (79.4 vs 68.4%, *P* = 0.048). Prognostic factors: ITS, age, days from onset to treatment Results of study in favor of MT
Filipo [156]	2012	ISSHL	PS	HBOT + IVS vs HBOT + ITS	< 15	No	NA	NA	NA	ITS better in patients with severe hearing loss (70–90 dB)
Imsuwansri [106]	2012	ISSHL	CR	SS + HBOT	36	Yes	NA	NA	NA	Case recovered completely after salvage HBOT
Alimoglu [99]	2011	ISSHL	RS	SS (A) vs HBOT (B) + SS (C) vs ITS vs HBOT (D)	≤ 3, ≤ 15 or > 15	No	NA	NA	HBOT(B): 42.6 SS: 19.0	Treatment started within 15 days was significantly better in each group Hearing gain was significantly higher in group B compared to A
Liu [105]	2011	ISSHL	RS	SS vs SS + DX vs SS + DX + HBOT	≤ 14	No	NA	NA	HBOT: 15.2 MT: 30.3	Significantly higher gain in the HBOT group when initial hearing loss ≥ 91 dB (*P* = 0.03)
Holy [130]	2011	ISSHL	RS	Methylprednisone + VD + HBOT	< 10, > 10	Yes	NA	NA	58.1	More hearing recovery when treated within 10 days
Körpinar [157]	2011	ISSHL	RS	HBOT monotherapy	11.2	Yes	29.5	NA	NA	Prognostic factors: early onset, high number HBOT sessions, steroid usage, profound hearing loss
Ohno [104]	2010	ISSHL	RS	SS + IVS (presalvage) SS + ATP + vitamins vs HBOT	7.4 weeks	Yes	HBOT: 5.2 MT: 2.0	HBOT: 8.2 MT: 3.5	HBOT: 2.0 MT: 0.0	Very modest effect of HBOT due to very late start of therapy
Lafère [103]	2010	AAT	RS	T1: SS + piracetam vs HBOT + T1 vs HBOT + IVS + piracetam IV	6–36 h	No	HBOT: 20.6 MT: 5.58	NA	NA	Early treatment was beneficial in the HBOT groups
Cekin [102]	2009	ISSHL	RCT	SS vs HBOT + SS + famotidine	≤3	No	NA	NA	HBOT: 55.3 MT: 42.9	Patients in the control group were more severely affected (95.9 dB vs 81.5 dB)
Ylikoski [78]	2008	AAT	RS	HBOT vs normobaric oxygen therapy	16.8	No	NA	HBOT: 69.3 NBOT: 56.2	NA	More benefit from hyperbaric conditions compared to normobaric conditions
Fujimura [88]	2007	ISSHL	RS	SS vs HBOT + SS	6.3	No	NA	HBOT: 64.4 MT: 56.0	HBOT: 17.9 MT: 25.4	Hearing improvement rate was significantly higher in the HBOT group when initial hearing loss was ≥ 80 dB
Dundar [87]	2007	ISSHL	PS	NA	NA	NA	NA	NA	HBOT: 38.2 MT: 12.0	
Satar [91]	2006	ISSHL	RS	MT: (vitamins + SS + DP piracetam) vs HBOT + MT	≤ 5	No	HBOT: 35.5 MT: 37.0	HBOT: 52.1 MT: 45.0	HBOT: 27.5 MT: 23.5	Percentage improved ears is higher in the MT group (76.4 vs 60%, *P* = 0.364)
Desloovere [86]	2006	ISSHL	RS	MT: IVS + PF + starch vs HBOT (1.5 ATA) vs HBOT (2.5 ATA)	HBOT (2.5): 30.8 HBOT (1.5): 54.3	Yes	HBOT (2.5): 19.7 MT: 2.6	HBOT(2.5): 25.9 MT: 8.0	HBOT(2.5): 41.0 MT: 6.3	Early treatment with HBOT was significantly better (*P* < 0.0001) Significantly higher gain after HBOT-2.5 (19.7 dB vs 2.6)
Horn [85]	2005	ISSHL	PS	HBOT	5.8 weeks	Yes	NA	NA	NA	No important differences
Winiarski [77]	2005	AAT	RS	Pharmacological treatment + HBOT	7	No	NA	NA	NA	Significant difference on 4.6 and 8 kHz when treatment was started within 5 days
Topuz [83]	2004	ISSHL	RCT	MT: SS, DX, DP, PF, salt restriction vs MT + HBOT	≤ 14	No	HBOT: 33.3 MT: 17.4	HBOT: 47.3 MT: 24.7	NA	Significantly higher hearing gains in HBTO patient when initial hearing loss greater than 61 dB
Racic [82]	2003	ISSHL	RS	IV PF vs HBOT	≤ 7	No	HBOT: 46.4 MT: 21.5	HBOT: 59.0 MT: 26.9	HBOT: 47.1 MT: 6.3	Significant hearing gains in the HBOT group (*P* = 0.001). After 9 months, 94.5% of HBOT patients had physiological hearing or mild hearing loss compared to 18.8% in the control group
Aslan [81]	2002	ISSHL	RS	MT BH + SS + SGB vs MT + HBOT	5.8	No	HBOT: 37.9 MT: 20.0	HBOT: 55.7 MT: 28.6	NA	In the HBOT group hearing gains were significantly higher in younger patients (< 50 years)
Inci [158]	2002	ISSHL	RS	HBOT	NA	Yes	9.7	12.8	3.9	
Fattori [133]	2001	ISSHL	RCT	Buflomedil (IV) vs HBOT	≤ 2	No	NA	HBOT: 61.3 MT: 24.0	HBOT: 56.7 MT: 25.0	Authors recommend HBOT for ISSHL
Nakashima [80]	1998	ISSHL	RS	Vitamin B, vasodilator, metabolic activators	NA	Yes	NA	NA	NA	HBOT within 1 week of onset of SSNHL symptoms was significantly better
Schwab [142]	1998	ISSHL	RS	Plasma expanders + PF vs HBOT	< 14	No	HBOT: 15.6 MT: 10.7	NA	NA	
Cavallazzi [101]	1996	ISSHL	RS	MT: SS + DX + vitamins + antiviral drugs + nicotinic acid + flunarizine vs MT + HBOT	NA	No	NA	NA	HBOT: 52.9 MT: 43.3	HBOT significantly better when treated within 3 days (95% vs 71%)
Vavrina [75]	1995	AAT	RS	Dextran + prednisone + HBOT vs control	< 3	No	HBOT: 121.3 MT: 74.3	NA	NA	
Dauman [159]	1993	ISSHL	PS	HBOT once or HBOT twice a day (both were combined with naftidrofuryl)	NA	No	NA	NA	NA	No difference between treating patients once or twice a day with HBOT
Zennaro [160]	1993	ISSHL	RS	Normovolemic dilution and HBOT	87	No	NA	NA	NA	Study obtained good results in more than half of the patients treated with HBOT
Pilgramm [76]	1985	AAT	PS	Sorbitol, dextran, betahistine + HBOT	NA	NA	NA	NA	NA	Beneficial effect of HBOT treatment (92% and 83% vs 87% and 62%)
Muzzi [89]	1984	ISSHL	RS	HBOT	<15, (15-30), > 30	Yes	8.6	16.0	NA	≥ 50 years had better hearing improvement
Demaertelaere [74]	1981	AAT	RS	NA	NA	NA	NA	NA	NA	
Goto [73]	1979	ISSHL	RS	MT (VD, SS, vitamins) vs HBOT + SGB vs MT + HBOT + SGB	NA	No	NA	NA	NA	All patients improved (10 dB) in the group with all the treatments combined
De Heyn [71]	1976	AAT	CR	VD vs VD and HBOT	NA	NA	NA	NA	NA	
Lamm [70]	1971	ISSHL	CR	NA	NA	NA	NA	NA	NA	
Appaix [69]	1970	ISSHL	CR	NA	NA	NA	NA	NA	NA	

*NA* not available, *DP* diazepam, *DX* dextran, *PF* pentoxifylline, *BH* betahistine, *SGB* stellate ganglion blocker, *VD* vasodilators, *LMWH* low-molecular weight heparin, *SS* systemic corticosteroids, *ITS* intratympanic corticosteroids, *IVS* intravenous corticosteroids, *RS* retrospective, *PS* prospective, *CR* case report

^a^Relative hearing gain has been calculated for studies by dividing the absolute hearing gain by the initial hearing loss. The studies were sorted by date of publication

### Intratympanic vs systemic corticosteroids

Alimoglu et al. found that systemic corticosteroids had significantly higher hearing gains and complete recovery (63.8% vs 46.5%) compared to intratympanic corticosteroids, when both drug delivery methods were combined with HBOT [99]. Sevil et al. compared two different steroid delivery strategies (intravenous and intratympanic) with HBOT for patients with ISSHL. They found no differences between the two delivery strategies. Naiboğllu et al. found that addition of intratympanic corticosteroids to HBOT with systemic corticosteroids does not result in better recovery [112].

So it seems that intratympanic delivery of corticosteroids does not have benefit over systemic corticosteroids. But, when systemic corticosteroids are contraindicated in a subgroup of patients, intratympanic corticosteroids can be considered as substitute therapy. In literature, the use of intratympanic steroids as first-line therapy for ISSHL was found to be superior to placebo therapy with intratympanic saline injections in a randomized, triple-blind, controlled trial [128].

### Early initiation of treatment

For acute losses, the acute initiation of therapy seems logical and many authors found that early treatment is beneficial. In AAT, Salihoglu et al. compared two groups: one group was treated within 10 days and the other one after 10 days. The early therapy initiation group was significantly better on 6, 8, 12.5, 14 and 16 kHz compared to the late initiated treatment group [129]. Lafère et al. found significantly higher hearing gains in the HBOT groups of patients suffering from AAT that started therapy within 6–43 h compared to the control group that received corticosteroid therapy within 48 h [103]. In line with these results, Bayoumy et al. [126] concluded that early initiation within 2 days of acoustic trauma with HBOT had significantly higher relative hearing gain compared to the same treatment started after 2 days.

Capuano et al. found that recovery was significantly better when patients were treated with HBOT in the first 14 days after ISSHL, than those treated after 14 days [100]. Holy et al. described that patients who were treated with HBOT within 10 days had significantly more improvement than those treated later than 10 days (66% vs 39%) [130].

Nakashima et al. reported that the final hearing level in patients treated with HBOT within 1 week from the onset of ISSHL was better than in those patients who were treated after 1 week [80].

Yildirim et al. found significantly higher gains in patients treated within 14 days after onset of hearing loss with HBOT, corticosteroids and piracetam, compared to patients who were treated after 14 days [110].

Hosokawa et al. reported significantly higher improvement rates in patients who were treated within 7 days (82.2%) of onset, compared to patients who were treated more than 7 days (42.7%, *P* < 0.001) after hearing loss [131].

Xie et al. showed that in patients who recovered from hearing loss, HBOT was initiated on average 5.6 days after onset of symptoms, whereas the non-recovered patients were treated on average after 9.1 days (*P* = 0.003) [120].

This highlights the importance of early intervention with HBOT. Therefore, it is recommended for both AAT and ISSHL that treatment with HBOT is started as early as possible.

An interesting side-step was published by Karatop-Cesur et al. who reported a negative correlation between the negative early treatment response (< 10 dB hearing gain in the first week) and failure of HBOT. In other words, when the hearing gain in the first week was less than 10 dB, then the probability of HBOT failure was higher [132].

### Severity of hearing loss

The severity of hearing loss may also play a role when choosing HBOT, as investigated by some authors. Fattori et al. obtained a significantly greater improvement in nine patients with severe hearing loss (> 70 dB) compared to ten patients with mild hearing loss (≤ 40 dB). Patients were treated within 48 h after the onset of hearing loss [133].

Topuz et al. reported that in the group (*n* = 10) with initial hearing level ≥ 81 dB and in the group who had hearing levels between 61 and 80 dB (*n* = 11), HBOT resulted in significantly higher hearing gains compared to their control groups (*n* = 4 and *n* = 11) who received corticosteroids, dextran, diazepam, pentoxifylline and salt restriction as treatment. However, there was no significant difference between the treatment and control groups when the initial hearing levels were ≤ 60 dB (6 control vs 13 HBOT patients). Treatment in this study was started within 2 weeks of the onset of hearing loss [83]. Furthermore, Fujimura et al. found significant hearing improvement rates following HBOT and corticosteroids (*n* = 24) compared to corticosteroids alone (*n* = 12) in patients with an initial hearing loss of ≥ 80 dB; this difference was not found when the initial hearing loss was smaller than 80 dB. In this study, treatment was started on average after 6.3 days [88].

Liu et al. had significantly higher hearing gain values in the HBOT group, which consisted of 46 patients with profound hearing loss (≥ 91 dB), compared to the control treatment. However, in severe (*n* = 35, 71–90 dB) and less severe (*n* = 31, ≤ 70 dB) hearing loss cases, this significant higher hearing gain difference was not found. Initiation of therapy occurred within 14 days after onset of hearing loss [105].

Furthermore, in 22 patients with hearing loss greater than 60 dB, Adjuk et al. reported significant hearing gains after salvage HBOT. These results were not so pronounced in patients with hearing loss smaller than 60 dB (21 patients). Patients were started with HBOT on average after 23 days from the end of steroid therapy. Steroid therapy was started after 11.4 days from the onset of ISSHL, thus 34.4 days were past between the onset of symptoms and HBOT [134].

The same results were obtained by Sherlock et al., who also found that patients with initial hearing loss greater than 60 dB (*n* = 44) had higher hearing gains than ≤ 60 dB (*n* = 34). HBOT was initiated on average 13 days after onset of ISSHL [135].

It may seem that HBOT has more beneficial effect when the hearing loss is more severe. However, this possibly just stems from the fact that the greater the loss, the more will be the effect demonstrated. Therefore, a calculation of the relative gain as described by Plontke et al. can be of great importance [136]. Another important factor might be that cases with more profound losses have lower spontaneous recovery levels and therefore treatment effects may be easier detectable or statistically significant and are more clinically relevant.

Contradictory results, however, were obtained by Cvorovic et al., who found significantly better hearing recovery in the intratympanic dexamethasone group compared to the salvage HBOT group when the initial hearing loss was ≥ 81 dB. Therapy was started within 4 weeks after the onset of ISSHL [137].

### Age

Sherlock et al. mentioned that patients younger than 50 years old had significantly higher hearing gains compared to patients older than 50 years (27 dB vs 19 dB) in a cohort of 78 HBOT patients [135].

Aslan et al. obtained significantly better results in the HBOT group in patients who were younger than 50 years (48.9 vs 14.5 dB, *P* < 0.001) [81].

Topuz et al. reported higher hearing gains in patients ≤ 50 years compared to those older than 50 years (39.1 vs 22.8 dB, *P* = 0.044) [83]. Furthermore, Cvorovic et al. also found significantly better hearing recovery in patients younger than 60 years old treated with HBOT (40.2 dB vs 21.2 dB) [137]. Edizer et al. obtained significant lower recovery in patients older than 60 years old [113].

Hosokawa et al. found significantly better improvement rates in patients who were 60 years old or younger (74.8%) compared to patients older than 60 years (62.9%, *P* = 0.024) [131].

Cekin et al. did not find any differences in hearing outcomes in patients younger or older than 50 years [102].

### Salvage therapy

Some studies used HBOT as a rescue or salvage therapy after failure of conventional therapy. Pezzoli et al. used HBOT as salvage therapy for patients who failed corticosteroid therapy. Although they found significant benefit for HBOT, these results were clinically very marginal [114]. Hosokawa et al. only obtained complete recovery rates of 9.6% and 6.2% in the HBOT and control groups, respectively, when HBOT was used as salvage therapy [138]. Ohno et al. found hearing gains of 5.2 dB and 2.0 dB in the HBOT and control group, respectively [104]. These patients received salvage HBOT therapy till 20 weeks after initial hearing loss. Sun et al. did not find any differences between the control, intratympanic dexamethasone and HBOT groups in the salvage treatment of ISSHL [139]. Horn et al. performed a prospective trial with HBOT in nine ISSHL patients who failed steroid and antiviral treatment. They started HBOT after an average of 5.8 weeks, and did not find any significant results [85].

Yang et al. found positive results of salvage combination therapy with HBOT and intratympanic corticosteroids. They reported good recovery in 68.4% of all patients treated with the salvage combination therapy, compared to salvage intratympanic corticosteroid monotherapy (48.6%), salvage HBO monotherapy (54.5%) and no salvage therapy (22.2%); the results were statistically significant (*P* = 0.018). Salvage therapy in this study was quickly initiated after failure of primary therapy. The combination therapy was initiated after 5 days from onset of hearing loss. Therefore, patients in this study were still in the theoretical plausible effective time frame of HBOT initiation [108].

Grounded on the theoretical foundation of HBOT and combined with these results, it is clear that the utilization of HBOT as salvage therapy is not the most effective option. HBOT is most likely to have good effects when it is used early after onset of symptoms, because it could prevent further ischemia within the inner ear in the time frame that the inner ear suffers most hypoxia.

### Other therapies

Due to the proposed vascular etiology, many other treatment modalities were proposed and experimentally tested. Examples of treatments are blood flow-promoting agents, vasodilators, diuretics, dextran and pentoxifylline. However, many of those studies (Chi, Liu, Ohno, Satar, Narozny, Aslan, Topuz, Racic) have failed to show effectiveness in the clinical setting [81–84, 91, 104, 105, 121].

### Recovery in recent systematic reviews

In 2014, van der Veen et al. published a review on the effectiveness of HBOT in AAT. Due to the small amount of studies and poor methodology of the studies, it was concluded that it was unclear what the clinical effect is of HBOT in AAT [140].

In ISSHL, spontaneous recovery ranges between 25 and 39% and most commonly occurs within the first 24 h [141]. In 2012, Bennet et al. published a Cochrane review, in which they proposed that HBOT may work for ISSHL but, due to the very low amount of studies (Topuz, Fattori, Schwab, Cavallazzi, Hoffmann, Pilgramm) [83, 101, 133, 142–144] with small patient sizes and poor methodology, it was not possible to draw strong conclusions. They reported that the mean difference in hearing gain between HBOT and control treatment was 15.6 dB (*P* = 0.039). Furthermore, they found that the recovery (> 25% return of hearing level) was in favor of HBOT [RR 1.39 (1.05–1.84), *P* = 0.022] [145].

More recently, Rhee et al. performed a meta-analysis on the complete recovery in HBOT and medical therapy (MT) patients. They found that in 14 studies, HBOT had significantly higher complete recovery (29.4% vs 20.6%, *P* = 0.03) compared to MT patients. However, it must be noted that the definition of complete recovery varied from study to study. Even though great heterogeneity exists between the definition of ‘complete recovery’ in different studies, HBOT was superior to MT in terms of complete recovery in the majority of studies (12 out of 14) [146]. Other authors (Eryigit, Saesen, Murphy-Lavoie) have also published reviews on the use of HBOT in ISSHL [27, 147, 148]. Their conclusions were mostly positive, especially showing results in favor of HBOT in patients with severe and profound hearing loss.

### Hyperbaric oxygen therapy

HBOT is a relatively time-consuming therapy for patients due to the fact that patients must be physically present in the oxygen chamber for 90 min in at least 10 days. Therefore, Attanasio et al. investigated whether addition of a second daily session of HBOT influenced the outcomes of treatment in ISSHL. They found no significant difference between one and two daily sessions; therefore, two daily sessions may be used for patients suffering from ISSHL [111]. This may improve therapy adherence and reduce the patients’ burden of therapy. HBOT is considered a safe therapy; however, side effects do occur in some studies. Most often side effects consist of barotrauma to middle ear and sinus [149]. Fujimura et al. reported side effects in 17 of the 67 patients (25.4%). Of these 17 patients, 9 developed otitis media with effusion, for which myringotomy was required in 4 cases, and 1 patient underwent tympanostomy tube insertion [88].

### No effect or negative results after HBOT

Gülüstan et al. found no significant difference between HBOT and ITS when compared to each other. This study only took into account both therapies as individual entities and did not combine both treatments as proposed in the rationale for therapy [116].

Tasdöven et al. reported higher levels of complete recovery in the group of patients who were treated with oral corticosteroids only compared to a group with combined oral corticosteroids and HBOT and another group consisting of oral corticosteroids and ozone therapy. However, the responses to therapy (hearing gain > 15 dB) was higher in the ozone and HBOT groups (82.4 and 61.5%) compared to the only corticosteroids group (50.8%) [150]. It was unclear what the time difference was from the onset of symptoms and initiation of therapy.

Almosnino et al. found no difference between the combination HBOT and ITS vs ITS in patients who failed on conventional steroid therapy. The reason for not finding a difference may be due to the late initiation of HBOT (29.1 days) [122].

### Recommendations

For future studies, we recommend starting therapy as early as possible, preferably within 48 h and to use combination therapy consisting of HBOT and corticosteroids. Furthermore, we recommend the use of standardized outcomes with absolute and relative hearing gains, especially in the affected frequencies and to collect speech recognition outcomes. For clinical recovery we refer to the guidelines of the American Academy [151].

## Conclusion

HBOT has been used quite extensively for acute hearing loss in the last couple of decades. This narrative review has described the rationale and clinical evidence for early initiation of HBOT combined with corticosteroids for, especially severe, acute hearing loss. Even though most studies were not randomized controlled trials, we think that based on the number of of studies showing a positive effect, HBOT should be discussed with patients (shared decision making) as optional therapy in case of AAT and ISSHL.
